# CArdiac and REspiratory adaptive Computed Tomography (CARE-CT): a proof-of-concept digital phantom study

**DOI:** 10.1007/s13246-022-01193-5

**Published:** 2022-11-25

**Authors:** Natasha Morton, Paul Keall, Ricky O’Brien, Tess Reynolds

**Affiliations:** 1 Faculty of Medicine and Health, University of Sydney, Sydney, NSW 2006 Australia; 2 School of Health and Biomedical Sciences, RMIT University, Melbourne, Australia

**Keywords:** CT, Radiotherapy, Motion management

## Abstract

Current respiratory 4DCT imaging for high-dose rate thoracic radiotherapy treatments are negatively affected by the complex interaction of cardiac and respiratory motion. We propose an imaging method to reduce artifacts caused by thoracic motion, CArdiac and REspiratory adaptive CT (CARE-CT), that monitors respiratory motion and ECG signals in real-time, triggering CT acquisition during combined cardiac and respiratory bins. Using a digital phantom, conventional 4DCT and CARE-CT acquisitions for nineteen patient-measured physiological traces were simulated. Ten respiratory bins were acquired for conventional 4DCT scans and ten respiratory bins during cardiac diastole were acquired for CARE-CT scans. Image artifacts were quantified for 10 common thoracic organs at risk (OAR) substructures using the differential normalized cross correlation between axial slices (ΔNCC), mean squared error (MSE) and sensitivity. For all images, on average, CARE-CT improved the ΔNCC for 18/19 and the MSE and sensitivity for all patient traces. The ΔNCC was reduced for all cardiac OARs (mean reduction 21%). The MSE was reduced for all OARs (mean reduction 36%). In the digital phantom study, the average scan time was increased from 1.8 ± 0.4 min to 7.5 ± 2.2 min with a reduction in average beam on time from 98 ± 28 s to 45 s using CARE-CT compared to conventional 4DCT. The proof-of-concept study indicates the potential for CARE-CT to image the thorax in real-time during the cardiac and respiratory cycle simultaneously, to reduce image artifacts for common thoracic OARs.

## Introduction

For more than a decade, respiratory 4-Dimensional Computed Tomography (4D CT) has supported the increasing uptake in high dose rate radiotherapy treatments, such as Stereotactic Body Radiation Therapy (SBRT), for thoracic tumor sites [1]. Unfortunately, 4D CT is limited in its capacity to simultaneously account for multiple motion sources, such as respiration and cardiac function, introducing image artifacts into the planning CT. This can affect machine learning auto segmentation accuracy [2] and restrict potential use cases for high dose rate radiotherapy where the target and organs at risk (OAR) undergo complex motion due to both cardiac deformation and respiration. Poor motion compensation combined with steep dose gradients can increase the risk of exceeding dose constraints in OARs, particularly where critical structures lie close to the target [3–8]. An example for cancer radiotherapy is the treatment of central lung or esophageal tumors located within close proximity to the heart [9–15]. Outside of cancer radiotherapy, an example is the novel treatment of cardiac arrhythmias using stereotactic radioablation as a non-invasive method of stopping irregular cardiac electric signals [16, 17]. In this case, treatment is delivered in one fraction where the heart itself is the target. In both examples, it is therefore pivotal that adequate motion compensation techniques are employed throughout treatment, beginning with pre-treatment imaging.

Current standard of care for lung radiotherapy imaging, respiratory 4D CT, acquires imaging data over the patient’s entire respiratory cycle but ignores the effects of cardiac motion, resulting in artifact prone images of and in proximity to the heart. These artifacts present as blurring and discontinuities in anatomical structures that lead to difficulty in accurately contouring and defining the range of motion for both the target and organs at risk [18–20]. Conversely, cardiac 4D CT acquires imaging data over the patient’s entire cardiac cycle but requires respiratory motion suppression techniques such as breath hold, abdominal compression, or single bin gating (imaging only during peak inhale or exhale) to reduce respiratory related artifacts. Similar techniques must then be utilized during treatment and have had variable success across patients [21–23].

Both cardiac and respiratory 4D CT rely on retrospective gating, where imaging data is oversampled and sorted into multiple image bins/phases post-scan, leading to wasted imaging dose [24–28]. Retrospective gating for combined respiratory-cardiac 4D CT requires long scan times with excessive oversampling to provide enough overlap between all cardiac and respiratory phase combinations for reconstruction. Prospective gating methods are common in both cardiac and respiratory imaging, acquiring during a specific phase, such as peak exhalation, based on a global displacement tolerance, or cardiac diastole, based on a set time after r-peak detection [29–33]. Combined prospective gating has been explored for one cardiac and one respiratory phase bin in small animal micro-CT by Badea et al. [34] and similarly in clinical dual source CT for pediatric patients by Goo [35]. Although useful in capturing a single snapshot of the thorax in time, neither study collects imaging data for the full range of cardiac and respiratory motion or outlines a process for doing so. Novel motion-compensation reconstruction techniques and combined retrospective-prospective gating have been explored for micro-CT in small animal studies [36–39] and standard clinical CBCT systems and but have yet to be applied to clinical fan-beam CT [40–42].

We propose a prospective CArdiac and REspiratory adaptive CT (CARE-CT) method that simultaneously accounts for full cardiac and respiratory motion in real-time. CARE-CT uses in-depth signal analysis to build on existing cine 4D CT technologies and prospective gating methods to accommodate combined cardiac ECG signals and free-breathing respiratory displacement, over multiple phases, within one scan. In this study we demonstrate that cardiac and full respiratory motion can be simultaneously accounted for during CARE-CT imaging in real-time, and will reduce artifacts in common thoracic OARs, compared to standard of care imaging technique, respiratory only 4D CT.

## Methods

CARE-CT is an in-house software that prospectively evaluates cardiac electrocardiogram (ECG) and respiratory motion signals to automatically trigger cine CT acquisition within user-defined phases. To assess CARE-CT’s ability to reduce motion artifacts compared to the current standard of care imaging technique, respiratory 4D CT, CT acquisition was simulated on the deformable digital Extended Cardiac and Torso (XCAT) phantom [43] for 19 patient-measured cardiac and respiratory signals. For the CARE-CT acquisitions, images were generated for 10 respiratory phases during cardiac diastole to demonstrate artifact reduction for common OARs in lung SBRT and stereotactic non-invasive cardiac radioablation treatment. Image analysis was conducted for 10 thoracic substructures and compared between CARE-CT and conventional respiratory 4D CT.

To demonstrate the ability of CARE-CT to account for multiple cardiac and respiratory phases at the same time and to provide an insight into the trade-off between data acquisition and imaging time, image-free simulations were conducted for combinations of 6, 8 and 10 respiratory bins and 1–5 cardiac bins.

### Conventional respiratory 4D CT protocol

**Fig. 1:**
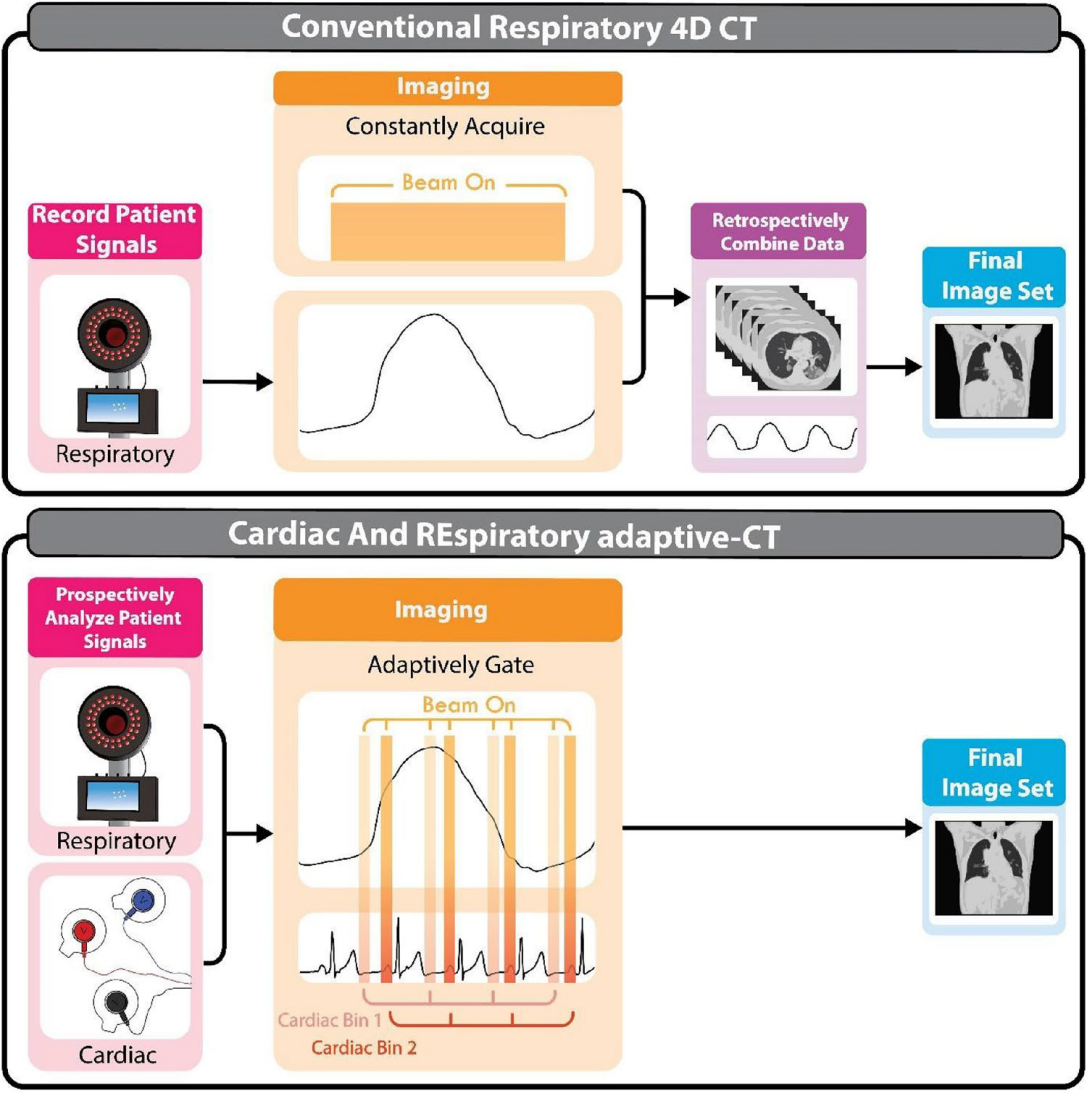
Comparison of conventional respiratory 4D CT and CArdiac and REspiratory adaptive CT

The current standard of care imaging for high dose rate lung radiotherapy treatments is respiratory 4D CT, referred to as conventional 4D CT throughout. [44, 45]. Here, to simulate conventional 4D CT, imaging data are continuously acquired for a pre-determined period of time before being retrospectively sorted into respiratory bins using the patient’s recorded respiratory signal, while cardiac information remains unaccounted for (Fig. 1). For the purposes of our simulation, we have followed the cine 4D CT protocol described by Pan, Lee, Rietzel, Chen [46] that proceeds as follows:

Respiratory data are monitored prior to imaging for *ttrain* = 30 s where an average breath length in seconds (*t̅r*) is determined. As imaging begins, acquisitions occur continuously for a period of *t̅r* + 1 s before a couch translation is triggered. Each scan is acquired over *Z* = 12 couch positions with a couch translation time of 1 s. Post-scan, the detected respiratory signal is analyzed and assigned a respiratory bin, *br* = 1, … *Br* where *Br* is the total number of respiratory bins, based on equidistant time through the respiratory cycle. Cardiac data are obtained for digital phantom simulation purposes but not used for CT acquisition triggering.

### CArdiac and REspiratory adaptive CT (CARE-CT) protocol

**Fig. 2:**
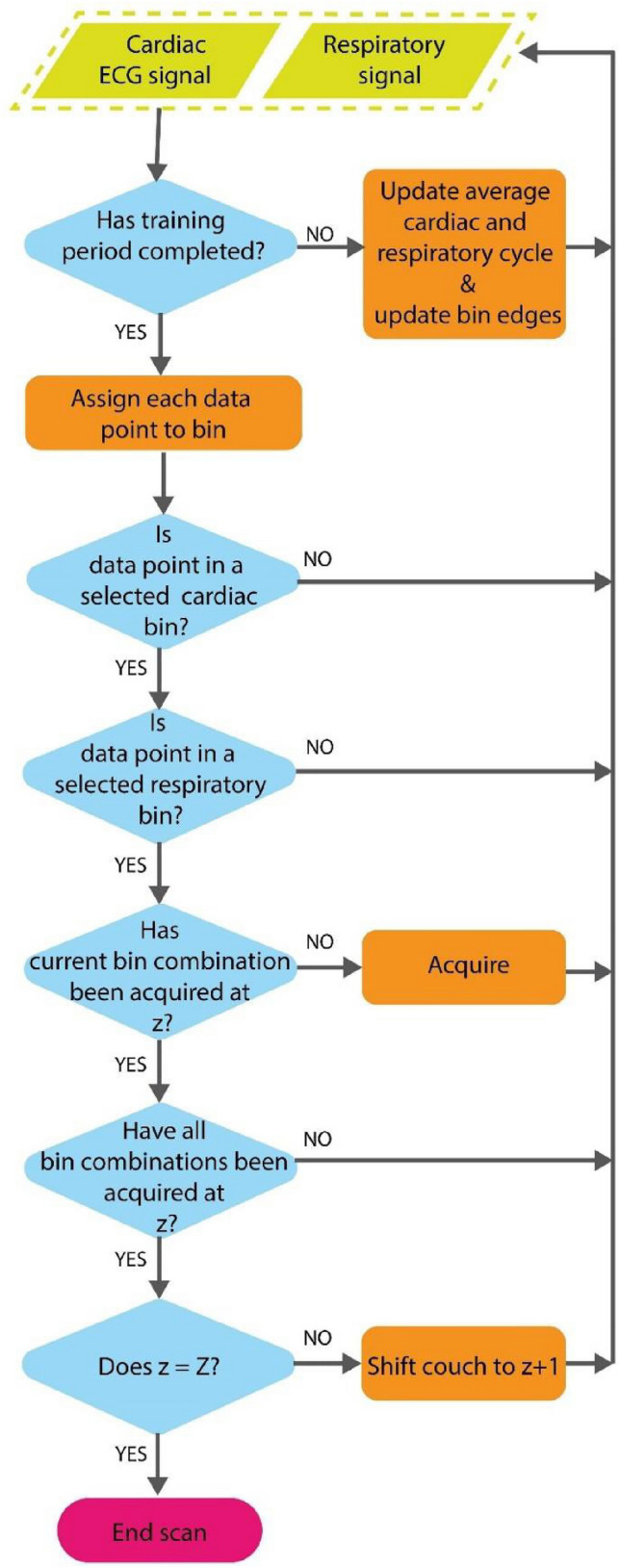
Prospective CARE-CT software workflow. The workflow is entered for each new incoming cardiac and respiratory signal

The CARE-CT protocol builds on the conventional respiratory 4D CT protocol through two main factors. The first is in the inclusion of a cardiac signal, used in conjunction with the respiratory signal, to drive the binning of imaging data. The second is in the switch from continuous CT acquisition, followed by post-scan data sorting, to adaptive gating, where the binning and data sorting occur mid-scan to drive cine CT acquisition (Fig. 1). The process for CARE-CT for the purposes of our simulation can be found in the graphical workflow, Fig. 2, and proceeds as follows:

Respiratory data are monitored prior to imaging for a training period of *ttrain* = 30 s where an average breath length in seconds (time between consecutive inhale peaks), *t̅r*, and an average cardiac cycle time (time between consecutive R-peaks), *t̅c*, are determined. As binning and data sorting needs to occur in real-time, during imaging, bin edges are defined post-training period and pre-imaging.

For both respiratory and cardiac binning, *t̅r* and *t̅c* are divided by the total number of respiratory bins, *Br*, and cardiac bins, *B*c, respectively, and used to define upper (*tU*_*br*_ and *tU*_*bc*_) and lower bin edges (*tL*_*br*_ and *tL*_*bc*_) for each respiratory bin, *br* = 1, … *Br* and cardiac *b*c = 1, … *B*c, as defined in Eqs. 1–4. This method was chosen to follow common binning approaches used in both respiratory and cardiac conventional 4DCT [24, 47].

1 $$tU_{br} = \left( {b_{r} + 1} \right)\frac{{\overline{{t_{r} }} }}{{B_{r} }}$$

2 $$tL_{br} = b_{r} \frac{{\overline{{t_{r} }} }}{{B_{r} }}$$

3 $$tU_{bc} = \left( {b_{c} + 1} \right)\frac{{\overline{{t_{c} }} }}{{B_{c} }}$$

4 $$tL_{bc} = b_{c} \frac{{\overline{{t_{c} }} }}{{B_{c} }}$$

As the training period completes, imaging will automatically commence. In order to trigger CT acquisition throughout imaging, the position of the current data point, *n*, in both the current respiratory and cardiac cycles must be calculated in real-time and corresponding bin values *br* and *bc* assigned. For respiration, the current position in the respiratory cycle is defined via a cyclical phase value between 0-2π, where peak inhale is defined as 0/2π and peak exhale as π. The phase values are determined through the application of the real-time signal processing method by Ruan *et al* [48]. The current position in the cardiac cycle is calculated based on the time since the previous R-peak and an average cardiac cycle time. The average cardiac cycle time is updated throughout imaging using a rolling average of the previous three cardiac cycles C.

T acquisition, for one gantry rotation, is triggered throughout the imaging process for each combination of respiratory and cardiac bins considered, at each couch position. If each combination of bins has been acquired at the current couch location, *z*, the couch translates to *z* + 1 and the acquisition process repeats. When the last couch position, *Z*, is reached, the scan concludes.

### Digital phantom: extended cardiac and torso (XCAT)

To simulate CT imaging on patient anatomy we employed the 4D extended cardiac-torso phantom (XCAT). The XCAT is a digital phantom software based on patient imaging data [43] and the Living Heart Model [49, 50]. The software simulates patient anatomy and physiology under motion, outputting a set of ‘patient’ volumes over time, thus, allowing multimodality imaging protocols to be emulated outside of the clinic. Several anatomical and physiological functions can be defined by user input. Of particular interest to this study is respiration, driven by diaphragm and chest displacements, and cardiac motion, driven by a percentage of respiratory displacement and cardiac phase.

The XCAT phantom has a predefined cardiac cycle based on the population average. This predefined motion deforms the heart to the same degree/volume every cardiac cycle. Consequently, variations in cardiac displacement from systole to diastole are not present. The user is, however, able to manipulate the XCAT cardiac motion, for each new data point, by providing it with a percentage through the cardiac cycle. Different percentages will deform the heart to different volumes based off the predefined XCAT cardiac cycle file.

**Fig. 3:**
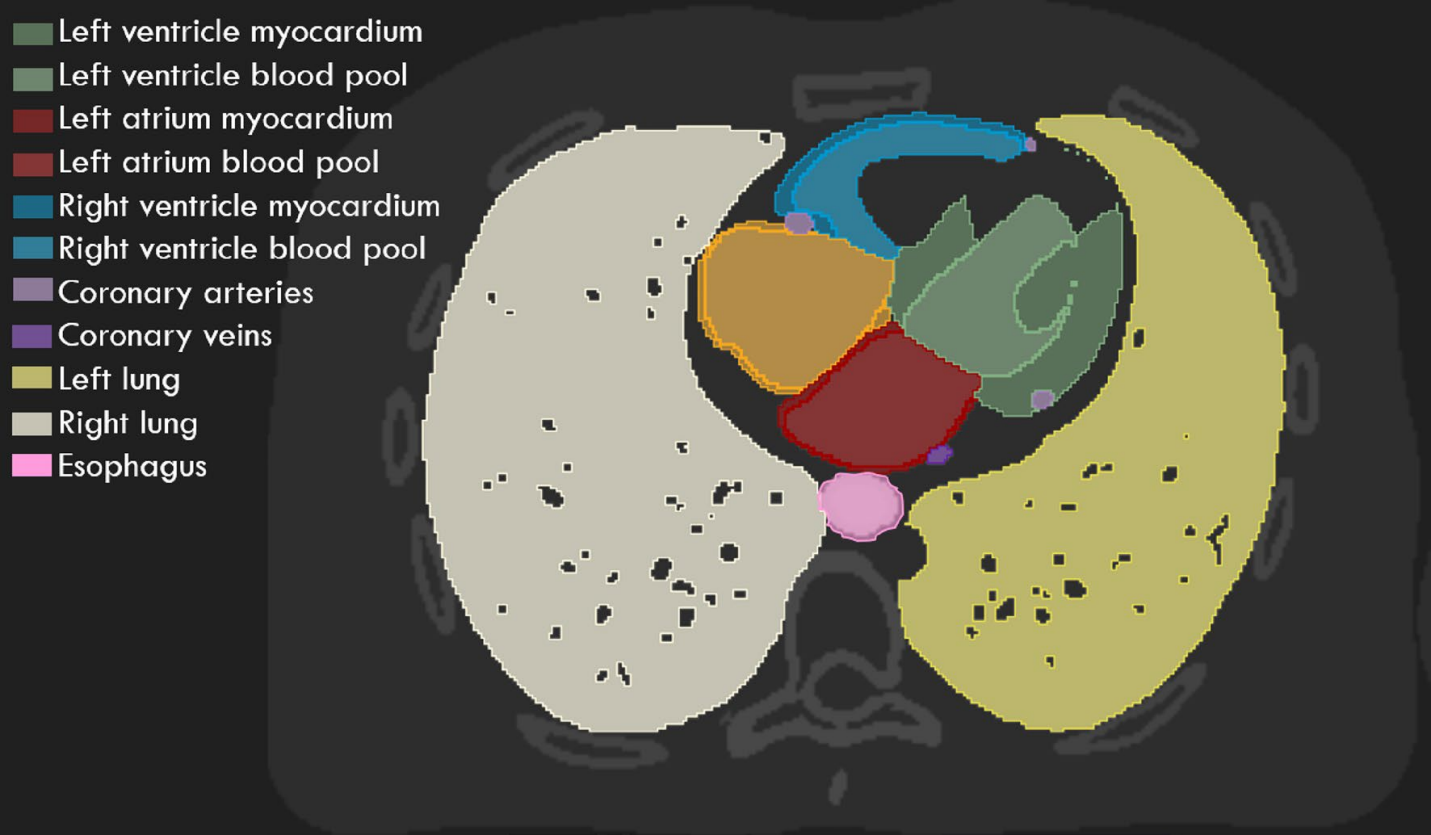
Axial slice view of the XCAT anatomy with 9 segmented thoracic structures that were used for artifact quantification

For ease of segmentation and to quantify the geometric accuracy of the imaging techniques, ten thoracic substructures within the XCAT phantom, as shown in Fig. 3, including the whole heart, four cardiac chambers, each lung, coronary arteries and veins, and the esophagus, were assigned differing and constant intensity values.

### Patient cardiac and respiratory traces

To program and deform the XCAT anatomy we utilized 19 patient-measured respiratory and cardiac traces. These traces were taken from the Combined ECG, Breathing and Seismocardiogram database (CEBS) [51] available from PhysioNet [52]. The database is comprised of 20 simultaneously acquired conventional ECG, free breathing respiratory and Seismocardiogram measurements from at-rest healthy volunteers. The measurements are split into three time recordings: an initial 5 min used to determine an incoming data baseline, 50 min while the volunteer listened to music, followed by a final 5 min sans-music. To avoid measurement variations at a no-music/music interface, the 50-min recording was used for this study (m001–m020). One patient signal set (m020) was excluded from this study due to noticeable interference of the ECG signal on the respiratory signal.

Respiratory data in the CEBS database were acquired using a piezoresistive band. The correlation between the band readings (mV) and chest or diaphragm motion in the database is unknown. As such, each respiratory trace was scaled between zero and a maximum peak-to-trough displacement value randomly sampled from a Students t-distribution. Metrics for the distribution were determined from Rit *et al* [53] who measured diaphragm motion during CBCT acquisition for 33 patients. In the Rit study, an average superior-inferior diaphragm displacement (peak-inhale to peak-exhale) of 16.4 mm and a standard deviation of 5.7 mm was determined. Final metrics for the 19 patient signal sets can be found in Table 1.

**Table 1:** Respiratory and cardiac signal metrics for 19 patients in the Combined Electrocardiograph, Breathing and Seismocardiogram database

Signal name	Resp. cycle time (s)		Resp. Displacement peak inhale-peak exhale (cm)		Baseline variation (cm)	Cardiac cycle time (s)	
	Mean	Std	Mean	Std	Drift	Mean	Std
M001	3.2	0.8	0.4	0.2	0.1	0.9	0.1
M002	3.4	0.4	1.1	0.2	– 0.2	0.9	0.1
M003	3.4	0.3	1	0.2	0	0.9	0
M004	3.1	0.4	1.5	0.4	0	0.9	0.1
M005	3.7	0.6	1	0.3	0	0.9	0.2
M006	4.2	1.3	0.7	0.4	– 0.1	1	0.1
M007	4.8	1.8	0.5	0.5	0.1	1.1	0.1
M008	3	0.8	0.6	0.1	0	0.6	0.1
M009	5.1	3.2	0.7	0.3	– 0.3	0.9	0.1
M010	3.7	0.5	0.8	0.2	0.1	1	0
M011	3.2	0.7	1.2	0.3	0	0.8	0
M012	2.6	0.4	0.9	0.1	0.1	0.8	0.1
M013	3.2	0.7	0.8	0.1	– 0.1	0.8	0.1
M014	4.2	0.9	0.4	0.4	0	0.9	0.1
M015	3.2	0.5	0.9	0.1	0	0.9	0.1
M016	3.5	1.1	0.8	0.2	0.1	0.8	0.1
M017	3.4	0.4	1.4	0.4	– 0.2	0.9	0.2
M018	4.4	2	0.7	0.4	0.1	0.7	0.1
M019	2.9	0.6	1	0.2	0	0.9	0.1

### Simulations

#### XCAT parameter

CT acquisitions using both the conventional 4D CT and CARE-CT protocols were simulated using the XCAT phantom. Each patient dataset outlined in “Patient cardiac and respiratory traces” was input into the CARE-CT software where a real-time decision on acquisition and couch translation was made. At the conclusion of a simulated scan, a series of XCAT volumes were generated based on the table position and cardiac and respiratory metrics recorded in the acquisition log file.

The first data point of each 0.36 s acquisition/beam on was used to simulate instantaneous x-ray acquisition. Instantaneous acquisition was selected to remove additional uncertainty from various reconstruction techniques and parameters and allow the acquisition technique to be the focus of this work. Scanner parameters were chosen to align with the Siemens Somatom Definition AS clinical scanner. Each acquisition corresponds to 2 cm of detector coverage, corresponding to 32 axial image slices. The diaphragm and chest wall are the two in-built points used to drive respiratory motion in the XCAT phantom. The patient measured respiratory trace was used as a displacement signal for both points, where the chest wall displacement was less than the diaphragm displacement ratio of 0.41 chest/diaphragm [54]. Cardiac motion due to respiration was also driven by the same patient measured respiratory traces in a ratio to 0.44 respiratory induced cardiac displacement/diaphragm [55].

The cardiac phase, used to drive the XCAT cardiac-induced heart motion, was determined from the retrospectively detected ECG signal where the time between adjacent R-Peaks was considered a complete cardiac cycle. The real-time cardiac phase, ∅*c*, was used to determine the points of acquisition, whereas the retrospective cardiac phase was used as a more robust measure to drive the motion of the XCAT phantom.

#### Scanner and protocol parameters

Conventional 4D CT and CARE-CT protocols were simulated with the following scanner parameters: gantry rotation of 0.36 s, 20 mm couch translations, 12 couch positions, 32 detector rows with individual detector widths of 0.625 mm and 1 mm^2^ transaxial resolution. For the conventional 4D CT, ten respiratory bins (*Br* = 10) were used. For the CARE-CT acquisitions, ten respiratory bins (*Br* = 10) were acquired during cardiac diastole (60%–80% through the cardiac cycle). Initially, as this is a proof-of-principle simulation study, only a single cardiac bin was selected to acquire all respiratory bins for the CARE-CT acquisition, where *Bc* = 5 and acquisition occurred for *bc* = 4. Cardiac diastole was chosen to encompass the heart at its largest left ventricular volume [56].

### Artifact quantification

The effects of respiratory and cardiac motion on image quality present as anatomical variations between adjacent axial slices, most notably at the junction between couch positions. Such variations, known as artifacts, can manifest in a number of ways, including duplication, overlapping (double structure discontinuities or elongation) or as incomplete structures [18]. The following three methods were chosen to detect artifacts of this nature and were calculated for 10 thoracic and cardiac structures segmented through intensity thresholding (Fig. 3).

1. The differential normalized cross correlation (*ΔNCC*), applied by Pollock [57], and based on the normalized cross correlation method by Cui. [58], was utilized. The normalized cross correlation was determined for pixel pairs across each adjacent axial slice using Eq. 5 where *br* is the 3D image set for each respiratory phase, *z* is the axial slice number, *I* is the pixel intensity and *x,y* denotes each pixel location. This defines regions of either similarity (+ 1) or dissimilarity (− 1) between slices. To obtain a total artifact measure of the entire bin volume (Δ*NCC*), the sum differences of the NCC at couch transition points, *n*, was taken as shown in Eq. 6.
  5 $$NCC\left({b}_{r},z\right)= \frac{\sum_{x,y}I({b}_{r},z)I({b}_{r},z+1)}{\sqrt{\sum_{x,y}{I({b}_{r},z)}^{2} \times \sum_{x,y}{I({b}_{r},z+1)}^{2} }}$$
  6 $$\Delta NCC\left({b}_{r}\right)= \sum_{n=1}^{12}\left|\frac{NCC\left({b}_{r},{z}_{n}-1\right)+NCC\left({b}_{r},{z}_{n}+1\right)}{2}-NCC({b}_{r},{z}_{n})\right|$$
2. The mean squared error, comparing the structures in the CARE-CT images to the corresponding ground truth images. The ground truth images were created by determining the mean cardiac phase, which implicitly determines the mean cardiac displacement, and respiratory displacement during acquisition for each patient dataset and bin combination. As such, each 3D image set, corresponding to a different respiratory phase bin, has a unique ground truth image for comparison.
3. Sensitivity of each protocol in correctly imaging each substructure, where the volume of overlapping regions between the CARE-CT images and the ground truth images (true positives, TP) was determined. Each overlapping volume was normalized in relation to the ground truth (TP + false negative (FN)).

To test whether there was a larger number of artifacts present in conventional 4D CT compared to CARE-CT, a one-tailed Wilcoxon signed rank test was used for each artifact metric.

### Effect of CARE-CT on scan time

Each additional bin that is acquired, whether respiratory or cardiac, is likely to lead to an increase in total scan time. To investigate the CARE-CT protocol for both data acquisition and imaging time, image-free CARE-CT simulations were completed using a combination of cardiac and respiratory bins. In this case, the simulations were conducted in real-time and were subject to the same scanner parameters as outlined earlier, however, the actual generation of XCAT images was suppressed.

For the respiratory bins, three total bin numbers, *Br* = 6, *Br* = 8 and *Br* = 10 were simulated where all bins, *br*, were acquired. For each of the total respiratory bins, a combination of cardiac bins from 1 to 5 were acquired where *Bc* = 5. This corresponded to a total of 15 scans for each patient trace.

## Results

**Fig. 4:**
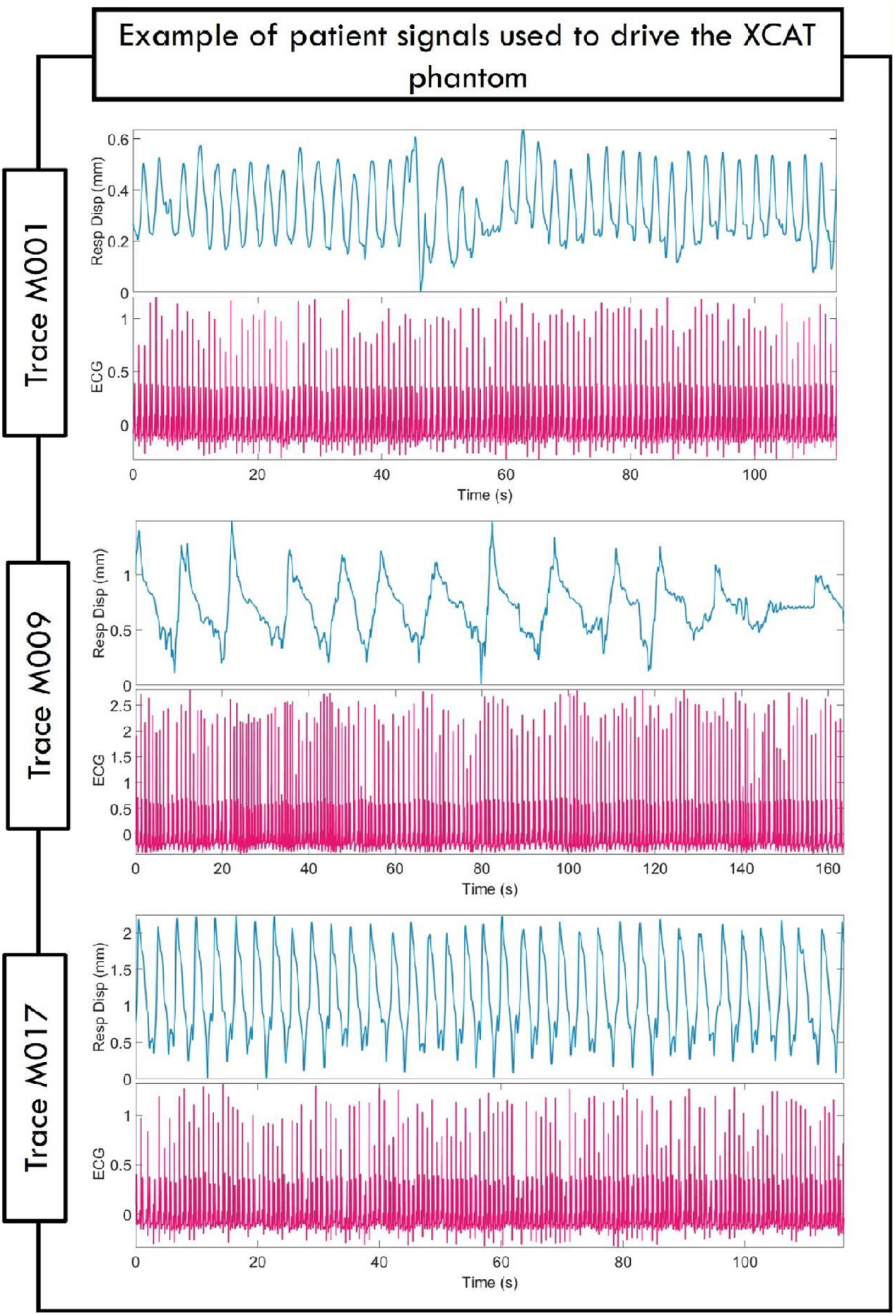
Example of three patient measured respiratory displacement and electrocardiogram signal used to drive the extended cardiac and torso (XCAT) phantom

**Fig. 5:**
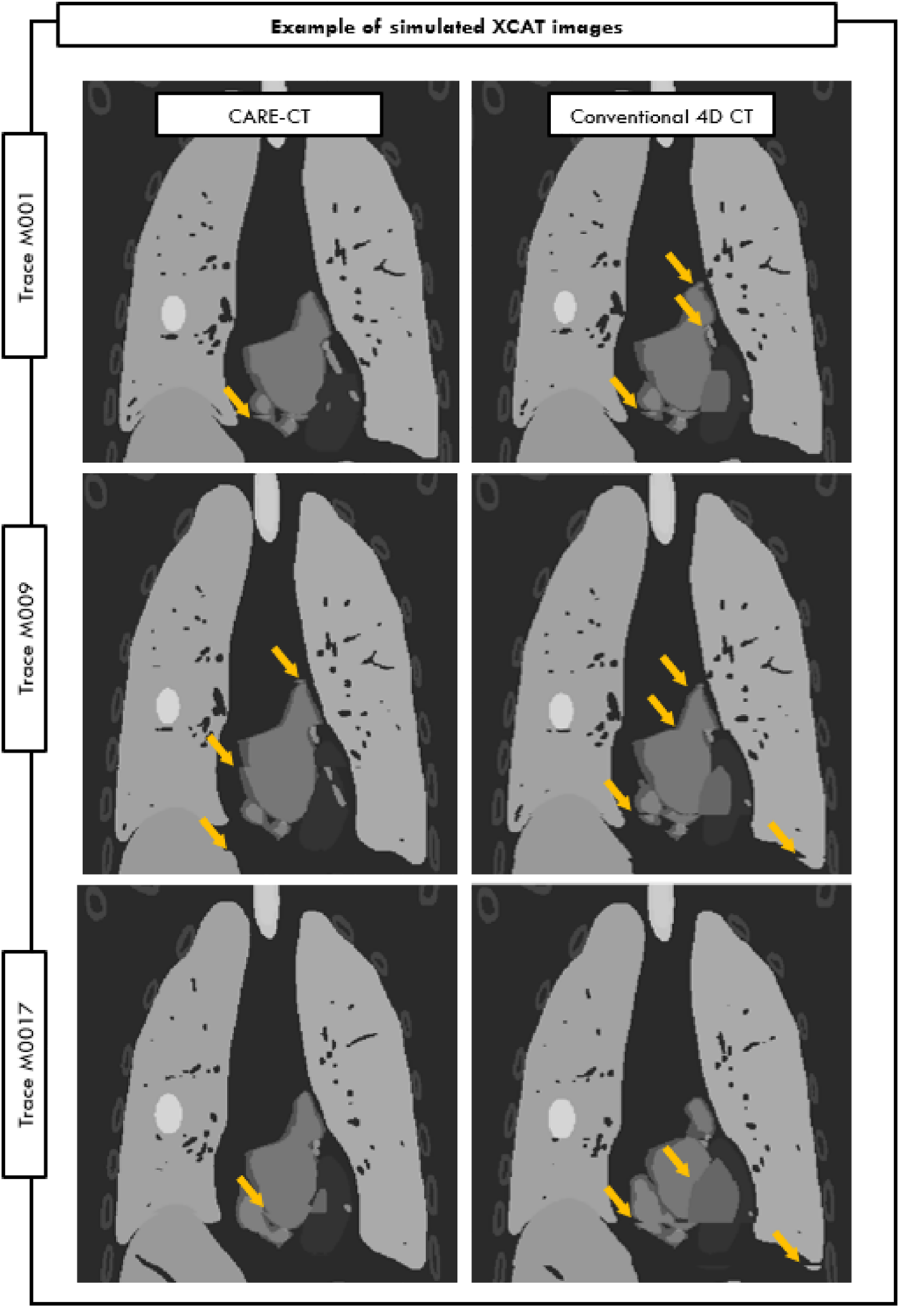
Coronal view of XCAT phantom for patient signal set M001, M009, and M017 during peak inhale. Image artifacts are highlighted by the orange arrows and can be seen in both the lungs and heart. Artifacts may continue along the same horizonal slice and can occur in multiple structures. Thoracic substructures were assigned individual and high intensity values for segmentation and are not representative of clinical CT values

An example of patient signals used to drive the XCAT phantom during image simulations is shown in Fig. 4 for patient signal sets M001, M009, and M017. For each of the three example patient signal sets, the corresponding XCAT images for peak inhalation are shown in Fig. 5. The images are taken from the 6 respiratory bin scans for both CARE-CT and conventional 4D CT.

### Normalized cross correlation

**Fig. 6:**
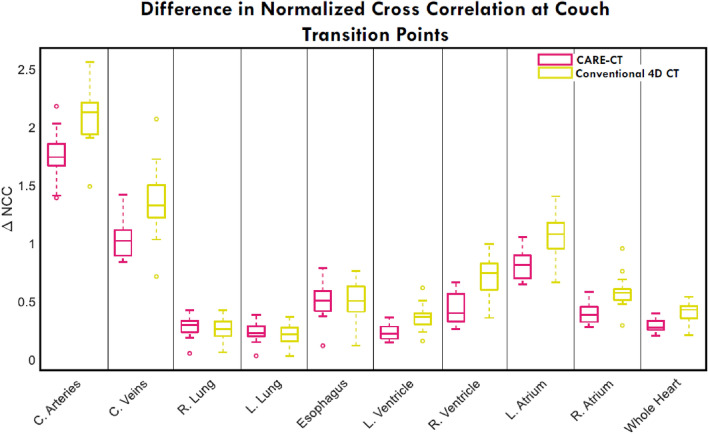
A comparison of the difference in normalized cross correlation at couch transition points for109 thoracic substructures defined in both conventional 4D CT and 10 respiratory bin CARE-CT scans. Values closer to zero denote fewer artifacts. Each box plot contains 19 data points, each representing the results for each patient trace (averaged across respiratory phase bins)

Δ*NCC* values for each thoracic structure can be found in Fig. 6. Overall, CARE-CT significantly reduced the pooled mean Δ*NCC* at couch transition points, compared to conventional 4D CT, by 21% (p = 0.01, determined by a one-tailed Wilcoxon signed rank test). The CARE-CT approach reduced Δ*NCC* for each of the cardiac chambers (range: 24% to 36%), the whole heart by 30%, the coronary arteries and veins by 17% and 24% respectively and increased the Δ*NCC* for the esophagus by 1% and the left and right lungs by 9% and 7% respectively. Focusing on the individual respiratory phase bins, the mean Δ*NCC*, pooled across each trace and thoracic structure, was reduced for all bins except for peak inhale (*br* = 10) which was increased by 1%. Peak exhale (*br* = 4) saw a reduction of 29%. The greatest reduction of 32% was for *br* = 5 and 6, where the equivalent conventional 4D CT bins had the largest Δ*NCC* of any phase bin. CARE-CT reduced Δ*NCC* for 18 of 19 patient traces.

### Mean square error

**Fig. 7:**
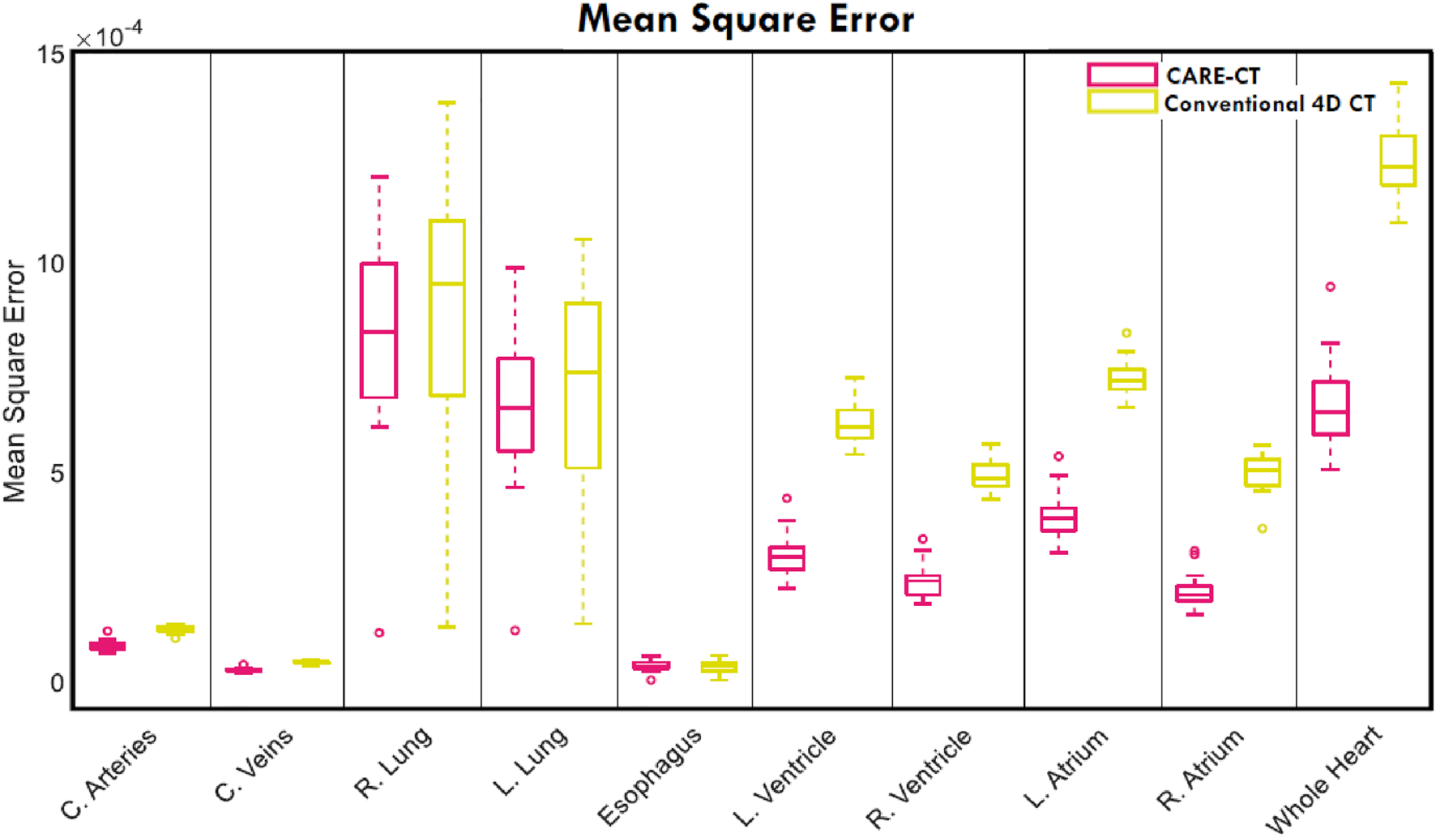
A comparison of the mean squared error in 9 thoracic substructures and the whole heart as compared to a ground truth for both conventional 4DCT and 10 respiratory bin CARE-CT scans. Values closer to zero denote a higher similarity between the ground truth and the moving image. Each box plot contains 19 data points, each representing the results for each patient trace (averaged across respiratory phase bins)

MSE values for each thoracic structure, averaged across phase bin, can be found in Fig. 7. Overall, CARE-CT significantly reduced the pooled mean MSE, compared to conventional 4D CT, by 36% (p = 0.002, determined by a one-tailed Wilcoxon signed rank test). The CARE-CT approach reduced MSE for each of the cardiac chambers (range: 46%–57%), the whole heart by 47%, the coronary arteries and veins by 30% and 39% respectively, the left and right lungs by 8% and 9% respectively and increased the MSE for the esophagus by 3%. Focusing on the individual respiratory phase bins, the mean MSE, pooled across each trace and thoracic structure, was reduced by 26% for peak inhale (*br* = 10) and 46% for peak exhale (*br* = 4). CARE-CT reduced MSE for all 19 patient traces.

### Overlapping volume

**Fig. 8:**
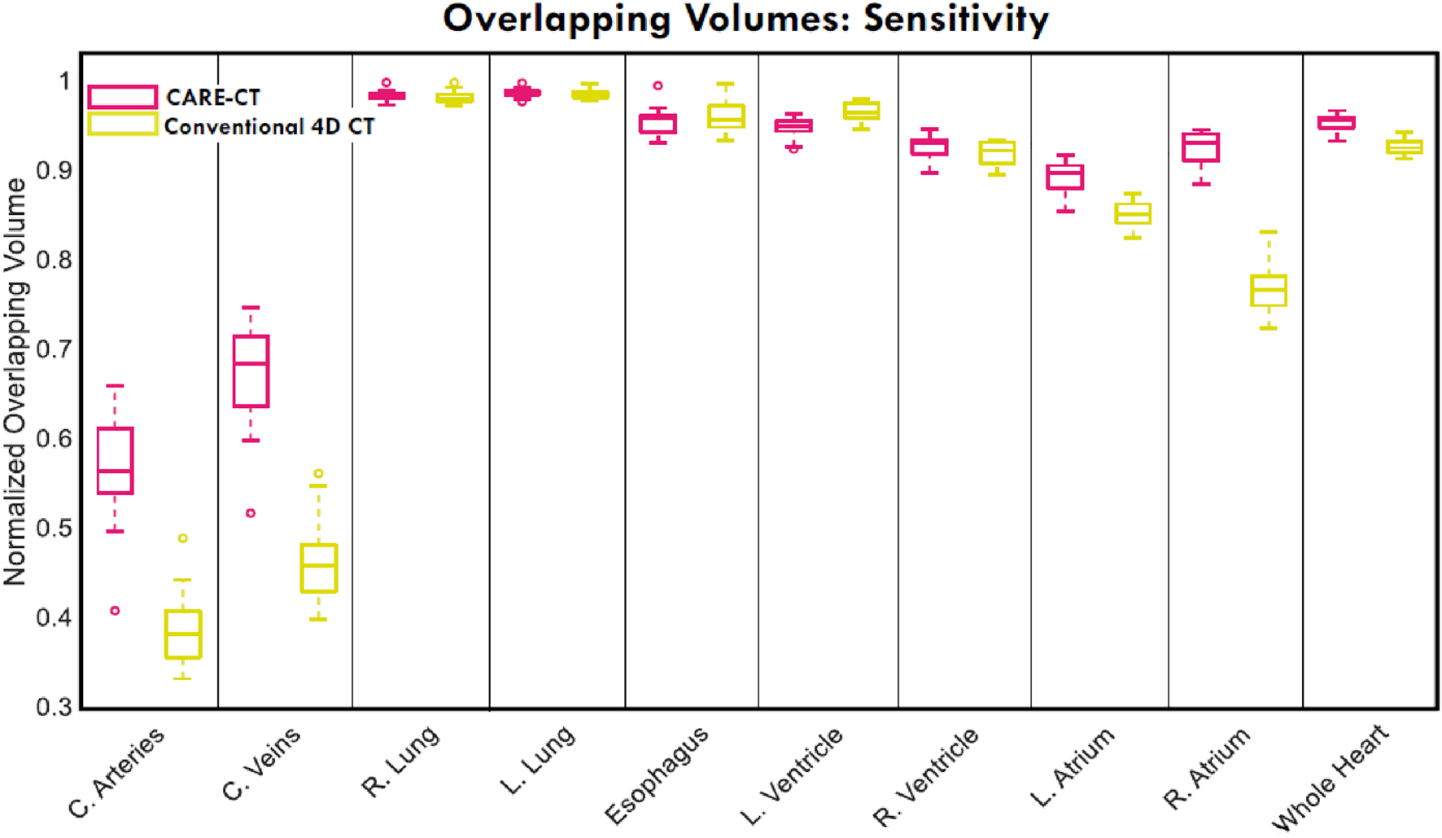
A comparison of the structure volumes overlapping the ground truth in 10 thoracic substructures for conventional 4DCT and 10 respiratory bin CARE-CT scans. A value of 1 denotes 100% structure overlap with the ground truth structures. Each box plot contains 19 data points, each representing the results for each patient trace (averaged across respiratory phase bins)

Overall, the structures in CARE-CT images were more closely overlapping with the ground truth compared to conventional 4D CT images (88% coverage versus 82% covered averaged across all structures considered), showing a significant difference of p = 0.02 (determined by a one-tailed Wilcoxon signed rank test). The results of the individual structures can be found in Fig. 8. CARE-CT showed an increase in coverage compared to conventional 4D CT for 6/10 thoracic structures and afforded the same coverage for 3/10 structures being the left and right lung and the esophagus. CARE-CT provided 2% less coverage compared to conventional 4D CT for the left ventricle. The greatest improvement was seen for the coronary veins where 21% more coverage was provided in the CARE-CT protocol. CARE-CT saw better structure overlap for all 19 patient traces.

### Total scan time

**Fig. 9:**
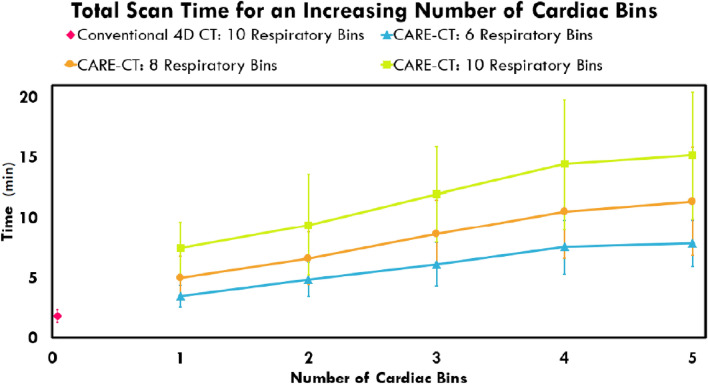
The effect of accounting for an increasing number of cardiac bins for the CARE-CT protocol on total scan time. The results display the average and standard deviation over all 19 traces. Total scan times for Conventional 4D CT (10 respiratory bins, 0 cardiac bins) are presented as a pink diamond. Total scans times for the CARE-CT XCAT simulations (10 respiratory bins, 1 cardiac bin) are presented as the first green square data point

The average scan times for the XCAT simulations (1 cardiac bin) can be seen in Fig. 9. Overall, the average total scan time was increased from 1.8 ± 0.5 min in the conventional 4D CT scans to 7.5 ± 2.2 min for the CARE-CT scans, where 16/19 scans were below 10 min.

The results of totals scan times for an increasing number of cardiac bins can be seen in Fig. 9. Increasing the number of cardiac bins led to an increase in scan times but at a decreasing rate.

### Beam on time

**Fig. 10:**
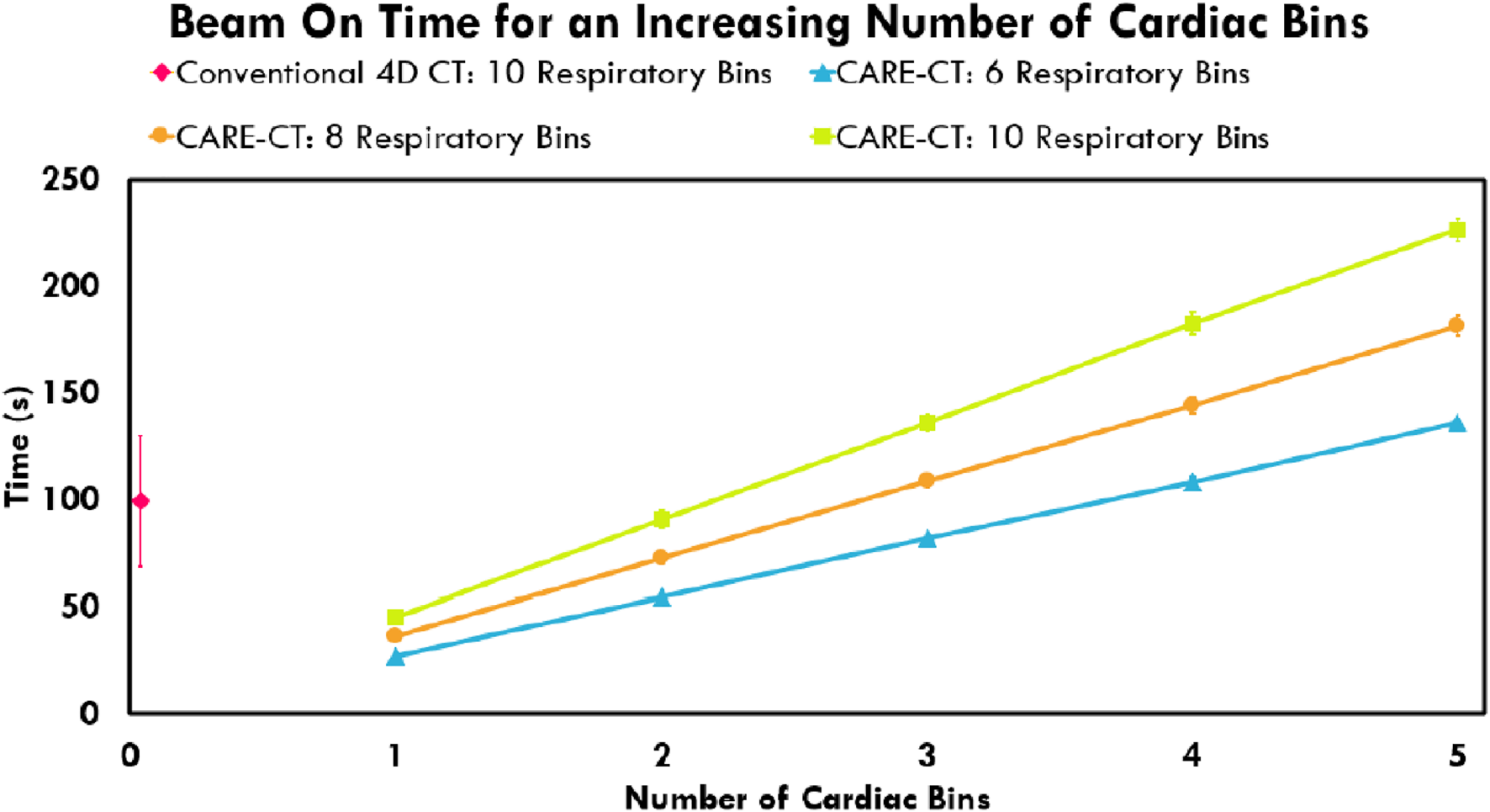
The effect of accounting for an increasing number of cardiac bins for the CARE-CT protocol on beam on time. The results display the average over all 19 traces. Each cardiac and respiratory bin combination for the CARE-CT scans will have the same beam on time across all 19 patient traces. Beam on times for Conventional 4D CT (10 respiratory bins, 0 cardiac bins) are presented as a pink diamond. Beam on times for the CARE-CT XCAT simulations (10 respiratory bins, 1 cardiac bin) are presented as the first green square data point

The average beam on times for the XCAT simulation scans can be found in Fig. 10. Overall, the average beam on time was reduced from 98.3 ± 28.1 s in the conventional 4D CT scans to 45 s for the CARE-CT scans, representing more efficient use of the imaging dose. The beam on time when acquiring for an increasing number of cardiac bins can be found in Fig. 10. This resulted in a linear increase per extra bin of 27 s for the 6 respiratory bin scans, 36 s for the 8 respiratory bin scans and 45 s for the 10 respiratory bin scans.

## Discussion

In this proof of principal study, we have demonstrated that combined cardiac and respiratory CT imaging can be achieved, providing a reduction in image artifacts for common thoracic OARs, particularly the cardiac substructures.

In radiation therapy, the whole heart is commonly delineated to encompass the cardiac structures and is used to help drive treatment planning. Currently, cardiac substructures individually are not a core guiding factor in cancer radiation therapy planning. Given recent studies linking chamber dose to toxicity [59–63], improved non-contrast semi-automatic and automatic chamber delineation techniques [2, 64–69] and the shift towards higher dose rate treatments, the cardiac chambers may become a higher priority in thoracic cancer radiation therapy planning. For techniques such as non-invasive cardiac radioablation where the heart itself is the target, dose to cardiac substructures is unavoidable and concerns over substructure toxicity will play a major role in treatment planning. As such, this study presents a promising basis to improve CT imaging for thoracic cancer and arrhythmic patients, where currently cardiac motion is unaccounted for.

The CARE-CT method additionally offers a potential saving in imaging dose compared to conventional respiratory 4D CT techniques. The reduction in beam-on time is due to the prospective selection of the cardiac and respiratory bins in the CARE-CT protocol compared to oversampling and retrospectively sorting data in conventional cases. Currently, CARE-CT does not share data across phase bins and as such, any increase in the number of bins (respiratory or cardiac) acquired will lead to an increase in the beam on time. In the current study, combinations up to 6 respiratory bins and 3 cardiac bins afforded less beam on time, on average, than conventional 4D CT, but reductions could be seen in individual cases with combinations of up to 6 respiratory bins and 5 cardiac bins, suggesting that scan times are heavily dependent on individual patient signals.

The current CARE-CT protocol has been designed with radiation therapy in mind where long 4D scan times are common, and contrast is rarely used. In this scenario CARE-CT is likely to be a good fit with deep learning based cardiac segmentation techniques to identify the radiation dose delivered to cardiac substructures. Additionally, as radiation therapy treatments take place over minutes, not seconds, a longer planning scan has the potential to better capture the representative motion of the patient during the subsequent treatment. However, for applications requiring the administration of a contrast agent, scan time reduction techniques will be needed.

We envisage the CARE-CT technique having the greatest impact if followed by a dual-gated treatment. Although, even for free-breathing radiotherapy, clear heart structures have the potential to provide a better basis for determining dose to structures close to or within the heart. However, systemic errors of the position of the structures could result in a misleading indication of structural dose. While the impact of cardiac induced image artifacts on radiotherapy treatment has not been widely studied, the impact of respiratory induced image artifacts on free-breathing radiotherapy treatment is known [20]. Given CARE-CT reduces artifacts in the cardiac region, it may allow for improved target and organ at risk internal target volumes (ITV), particular in stereotactic arrhythmic radiotherapy treatments where the radiation beam is aimed at the heart itself.

This study is purely a proof-of-concept and as such, CARE-CT has not been optimized to specific conditions or end points (scan time v dose v image quality). In a clinical setting, optimization between the amount of acquired imaging data and total scan time will need to be explored and is likely to differ on an application-to-application basis. The combination of a high frequency cardiac cycle with low frequency respiratory cycle provides fewer opportunities for dual-gated acquisition and becomes more challenging with the inclusion of patient-specific signal variations. An increase in scan time was demonstrated not only when comparing CARE-CT to conventional 4D CT, but within CARE-CT itself where 6 respiratory bins and one cardiac bin was four times shorter than 10 respiratory bins and 5 cardiac bins. The scan time may be reduced through multi-segment or half-rotation reconstruction techniques (often used in cardiac imaging), allowing multiple cardiac phases to be captured within each acquisition. Specialized hardware such as dual-source CT scanners additionally reduce the effective gantry rotation time [70–72], increasing the number of cardiac phases that can be completely captured in a single rotation for a larger range of heart rates. These techniques have the added benefit of reducing blur artifacts often present in the cardiac region when large or fast cardiac motion is present during the finite gantry rotation/acquisition time. Such motion has long been a limitation of cardiac imaging, particularly for patients with a high heart rate where systolic phases in the cardiac cycle may be complete within a fraction of the time taken for an acquisition of ≥ 360 ms.

A limitation of this study is the simulation of instantaneous acquisition, chosen to remove the added uncertainty from various reconstruction techniques and parameters, leading to blur-free slices in both the CARE-CT and conventional 4D CT cases. As such, the results presented in this study represent an upper limit in the achievable image quality improvement of dual respiratory and cardiac motion corrected CT image acquisition.

Our data source was limited to 19 patients which may not represent the full variation observed clinically. The XCAT phantom was anatomically the same across all scans with the same motion models and correlation between diaphragm:chest wall and diaphragm:cardiac motion (respiratory component) used for each simulation. Due to the lack of synchronously acquired ECG and respiratory motion databases, the current study uses signals from healthy patients. Before clinical implementation, further research and development should be made into determining the accuracy of CARE-CT in unhealthy patients where greater variations in both signals are more likely to occur. For example, this study uses a rudimentary R-peak detection method that works well for the current example but may struggle in accuracy with the introduction of arrhythmic cycles, likely to occur in patients being treated with non-invasive stereotactic radioablation. Conversely, the respiratory signal uses a rigorously tested phase prediction method by Ruan et al. [48] but fails to account for large variations in the patient’s breathing signal, likely to occur in thoracic cancer patients who struggle to breath with ease. To an extent, the effects of respiratory variations on artifacts can already be seen in the results of this study. The first, is the consistent underperformance of CARE-CT to significantly reduce artifacts in the lungs compared to cardiac substructures, where the lungs, as a large structure, will feel greater effects of respiration over cardiac function. The second, is the deviation in results when comparing peak inhale to peak exhale, where peak inhale, known for its instability across respiratory cycles, displayed a lower reduction in artifacts compared to peak exhale. In both cases, respiratory signal changes are likely to induce image artifacts. As CARE-CT is a prospective gating protocol, it may be possible to avoid breathing irregularities by pausing imaging until such irregularities or variations pass, as implemented in the REACT software73,74. Increased beam pausing will likely result in increased scan times, to the extent of which will be patient dependent. Should this method be adopted, optimization between signal irregularity compensation and total scan times will need to be investigated.

While the CARE-CT protocol requires further testing and development to demonstrate the viability for clinical translation, this study has demonstrated the feasibility of reducing motion artifacts through prospectively gating combined cardiac and free breathing respiratory signals. Efforts should be made to evaluate the efficacy of CARE-CT for application specific conditions, as well as reduce the total scan time while maintaining image quality, and where reduction in scan time cannot be achieved, patient tolerance should be assessed. CARE-CT has the added possibility of imaging dose reduction and with its similarity to current 4D CT set up and acquisition, CARE-CT should allow for relatively easy adoption into the clinical workflow.

## Conclusion

A method to prospectively account for combined cardiac and respiratory motion during pre-treatment CT imaging to reduce motion artifacts, called CARE-CT, has been proposed. During a digital phantom proof-of-concept study, CARE-CT demonstrated a significant reduction in image artifacts in cardiac substructures and reduced the beam on time when compared to conventional respiratory 4D CT.

## Data Availability

The data that support the findings of this study are available from the authors, upon reasonable request.

## References

[1] Holmes J, Zagar T, Chen R (2017). Adoption of stereotactic body radiotherapy for stage IA non-small cell lung cancer across the United States. JNCI cancer spectrum.

[2] Finnegan R, Dowling J, Koh ES (2019). Feasibility of multi-atlas cardiac segmentation from thoracic planning CT in a probabilistic framework. Phys Med Biol.

[3] Chen T, Qin S, Xu X, Jabbour SK, Haffty BG, Yue NJ (2014). Frequency filtering based analysis on the cardiac induced lung tumor motion and its impact on the radiotherapy management. Radiother Oncol.

[4] Zou W, Yin L, Shen J (2014). Dynamic simulation of motion effects in IMAT lung SBRT. Radiat Oncol.

[5] Kry SF, Bednarz B, Howell RM (2017). AAPM TG 158: Measurement and calculation of doses outside the treated volume from external-beam radiation therapy. Med Phys.

[6] Savanovic M, Strbac B, Jaros D, Cazic D, Foulquier JN (2020). Impact of lung tumor motion on dose delivered to organ at risk in lung stereotactic body radiation therapy. J Radiat Oncol.

[7] Nardone V, Giugliano FM, Reginelli A (2020). 4D CT analysis of organs at risk (OARs) in stereotactic radiotherapy. Radiother Oncol.

[8] Mahadevan A, Emami B, Grimm J (2020). Potential clinical significance of overall targeting accuracy and motion management in the treatment of tumors that move with respiration: lessons learnt from a quarter century of stereotactic body radiotherapy from dose response models. Front Oncol.

[9] Chang JY, Bezjak A, Mornex F, Committee IART (2015). Stereotactic ablative radiotherapy for centrally located early stage non-small-cell lung cancer: what we have learned. J Thorac Oncol.

[10] Haseltine JM, Rimner A, Gelblum DY (2016). Fatal complications after stereotactic body radiation therapy for central lung tumors abutting the proximal bronchial tree. Pract Radiat Oncol.

[11] Nguyen KNB, Hause DJ, Novak J, Monjazeb AM, Daly ME (2019). Tumor Control and toxicity after SBRT for ultracentral, central, and paramediastinal lung tumors. Pract Radiat Oncol.

[12] Thompson M, Rosenzweig KE (2019). The evolving toxicity profile of SBRT for lung cancer. Transl Lung Cancer Res.

[13] Palmer J, Yang J, Pan T, Court LE (2014). Motion of the esophagus due to cardiac motion. PLoS ONE.

[14] Hayashi Y, Iijima H, Isohashi F (2019). The heart's exposure to radiation increases the risk of cardiac toxicity after chemoradiotherapy for superficial esophageal cancer: a retrospective cohort study. BMC Cancer.

[15] Garant A, Spears G, Routman D (2021). A multi-institutional analysis of radiation dosimetric predictors of toxicity after trimodality therapy for esophageal cancer. Pract Radiat Oncol.

[16] Knutson NC, Samson PP, Hugo GD (2019). Radiation therapy workflow and dosimetric analysis from a phase 1/2 trial of noninvasive cardiac radioablation for ventricular tachycardia. Int J Radiat Oncol Biol Phys.

[17] Wei C, Qian P, Tedrow U, Mak R, Zei PC (2020). Non-invasive stereotactic radioablation: a new option for the treatment of ventricular arrhythmias. Arrhythm Electrophysiol Rev.

[18] Yamamoto T, Langner U, Loo BW, Shen J, Keall PJ (2008). Retrospective analysis of artifacts in four-dimensional CT images of 50 abdominal and thoracic radiotherapy patients. Int J Radiat Oncol Biol Phys.

[19] Persson GF, Nygaard DE, Brink C (2010). Deviations in delineated GTV caused by artefacts in 4DCT. Radiother Oncol.

[20] Sentker T, Schmidt V, Ozga AK (2020). 4D CT image artifacts affect local control in SBRT of lung and liver metastases. Radiother Oncol.

[21] Mampuya WA, Nakamura M, Matsuo Y (2013). Interfraction variation in lung tumor position with abdominal compression during stereotactic body radiotherapy. Med Phys.

[22] Rasheed A, Jabbour SK, Rosenberg S (2016). Motion and volumetric change as demonstrated by 4DCT: The effects of abdominal compression on the GTV, lungs, and heart in lung cancer patients. Pract Radiat Oncol.

[23] Van Gelder R, Wong S, Le A (2018). Experience with an abdominal compression band for radiotherapy of upper abdominal tumours. J Med Radiat Sci.

[24] Desjardins B, Kazerooni EA (2004). ECG-Gated Cardiac CT. Am J Roentgenol.

[25] Castillo SJ, Castillo R, Castillo E (2015). Evaluation of 4D CT acquisition methods designed to reduce artifacts. J Appl Clin Med Phys.

[26] Vedam SS, Keall PJ, Kini VR, Mostafavi H, Shukla HP, Mohan R (2003). Acquiring a four-dimensional computed tomography dataset using an external respiratory signal. Phys Med Biol.

[27] Woodhouse CE, Janowitz WR, Viamonte M (1997). Coronary arteries: retrospective cardiac gating technique to reduce cardiac motion artifact at spiral CT. Radiology.

[28] Kachelriess M, Kalender WA (1998). Electrocardiogram-correlated image reconstruction from subsecond spiral computed tomography scans of the heart. Med Phys.

[29] Maruyama T, Takada M, Hasuike T, Yoshikawa A, Namimatsu E, Yoshizumi T (2008). Radiation dose reduction and coronary assessability of prospective electrocardiogram-gated computed tomography coronary angiography: comparison with retrospective electrocardiogram-gated helical scan. J Am Coll Cardiol.

[30] Hsieh J, Londt J, Vass M, Li J, Tang X, Okerlund D (2006). Step-and-shoot data acquisition and reconstruction for cardiac x-ray computed tomography. Med Phys.

[31] Giraud P, Yorke E Fau - Ford EC, Ford Ec Fau - Wagman R, et al. Reduction of organ motion in lung tumors with respiratory gating. (0169–5002 (Print)). 10.1016/j.lungcan.2005.08.008 16198022

[32] Keall PJ, Kini Vr Fau - Vedam SS, Vedam Ss Fau - Mohan R, Mohan R. Potential radiotherapy improvements with respiratory gating. (0158–9938 (Print)). 10.1007/BF03178368 12049470

[33] Ford EC, Mageras Gs Fau - Yorke E, Yorke E Fau - Rosenzweig KE, Rosenzweig Ke Fau- Wagman R, Wagman R Fau - Ling CC, Ling CC. Evaluation of respiratory movement during gated radiotherapy using film and electronic portal imaging. (0360–3016(Print)). 10.1016/s0360-3016(01)02681-5 11872300

[34] Badea C, Hedlund LW, Johnson GA (2004). Micro-CT with respiratory and cardiac gating. Med Phys.

[35] Goo HW (2018). Combined prospectively electrocardiography- and respiratory-triggered sequential cardiac computed tomography in free-breathing children: success rate and image quality. Pediatr Radiol.

[36] Brehm M, Sawall S, Maier J, Sauppe S, Kachelrieß M (2015). Cardiorespiratory motion- compensated micro-CT image reconstruction using an artifact model-based motion estimation. Med Phys.

[37] Cao G, Burk LM, Lee YZ (2010). Prospective-gated cardiac micro-CT imaging of free-breathing mice using carbon nanotube field emission X-ray. Med Phys.

[38] Badea CT, Schreibmann E, Fox T (2008). A registration based approach for 4D cardiac micro-CT using combined prospective and retrospective gating. Med Phys.

[39] Guo X, Johnston SM, Qi Y, Johnson GA, Badea CT (2012). 4D micro-CT using fast prospective gating. Phys Med Biol.

[40] Sera T, Yokota H, Fujisaki K (2008). Development of high-resolution 4D in vivo-CT for visualization of cardiac and respiratory deformations of small animals. Phys Med Biol.

[41] Kuntz J, Dinkel J, Zwick S (2010). Fully automated intrinsic respiratory and cardiac gating for small animal CT. Phys Med Biol.

[42] Sauppe S, Hahn A, Brehm M, Paysan P, Seghers D, Kachelrieß M (2016). Five-dimensional motion compensation for respiratory and cardiac motion with cone-beam CT of the thorax region. SPIE Med Imag.

[43] Segars WP, Sturgeon G, Mendonca S, Grimes J, Tsui BM (2010). 4D XCAT phantom for multimodality imaging research. Med Phys.

[44] Sahgal A, Roberge D, Schellenberg D (2012). The Canadian Association of Radiation Oncology scope of practice guidelines for lung, liver and spine stereotactic body radiotherapy. Clin Oncol (R Coll Radiol).

[45] De Ruysscher D, Faivre-Finn C, Moeller D (2017). European Organization for Research and Treatment of Cancer (EORTC) recommendations for planning and delivery of high-dose, high precision radiotherapy for lung cancer. Radiother Oncol.

[46] Pan T, Lee TY, Rietzel E, Chen GT (2004). 4D-CT imaging of a volume influenced by respiratory motion on multi-slice CT. Med Phys.

[47] Li H, Noel C, Garcia-Ramirez J (2012). Clinical evaluations of an amplitude-based binning algorithm for 4DCT reconstruction in radiation therapy. Med Phys.

[48] Ruan D, Fessler JA, Balter JM, Keall PJ (2009). Real-time profiling of respiratory motion: baseline drift, frequency variation and fundamental pattern change. Phys Med Biol.

[49] Segars WP, Veress AI, Sturgeon GM, Samei E (2019). Incorporation of the Living Heart Model into the 4D XCAT Phantom for Cardiac Imaging Research. IEEE Trans Radiat Plasma Med Sci.

[50] Baillargeon B, Rebelo N, Fox DD, Taylor RL, Kuhl E (2014). The Living Heart Project: a robust and integrative simulator for human heart function. Eur J Mech A Solids.

[51] García-González MA, Argelagós-Palau A, Fernández-Chimeno M, Ramos-Castro J (2013) A comparison of heartbeat detectors for the seismocardiogram. Paper presented at: Computing in Cardiology. 22-25 Sept. 2013, 2013

[52] Goldberger A, Amaral L, Glass L (2000). PhysioNet.

[53] Rit S, van Herk M, Zijp L, Sonke JJ (2012). Quantification of the variability of diaphragm motion and implications for treatment margin construction. Int J Radiat Oncol Biol Phys.

[54] Wade OL (1954). Movements of the thoracic cage and diaphragm in respiration. J Physiol.

[55] McLeish K, Hill DL, Atkinson D, Blackall JM, Razavi R (2002). A study of the motion and deformation of the heart due to respiration. IEEE Trans Med Imaging.

[56] Clay S, Alfakih K, Radjenovic A, Jones T, Ridgway JP, Sinvananthan MU (2006). Normal range of human left ventricular volumes and mass using steady state free precession MRI in the radial long axis orientation. MAGMA.

[57] Pollock S, Kipritidis J, Lee D, Bernatowicz K, Keall P (2016). The impact of breathing guidance and prospective gating during thoracic 4DCT imaging: an XCAT study utilizing lung cancer patient motion. Phys Med Biol.

[58] Cui G, Jew B, Hong JC, Johnston EW, Loo BW, Maxim PG (2012). An automated method for comparing motion artifacts in cine four-dimensional computed tomography images. J Appl Clin Med Phys.

[59] Stam B, Peulen H, Guckenberger M (2017). Dose to heart substructures is associated with non-cancer death after SBRT in stage I-II NSCLC patients. Radiother Oncol.

[60] Wong OY, Yau V, Kang J (2018). Survival impact of cardiac dose following lung stereotactic body radiotherapy. Clin Lung Cancer.

[61] Tang S, Otton J, Holloway L (2019). Quantification of cardiac subvolume dosimetry using a 17 segment model of the left ventricle in breast cancer patients receiving tangential beam radiotherapy. Radiother Oncol.

[62] Hoppe BS, Bates JE, Mendenhall NP (2020). The meaningless meaning of mean heart dose in mediastinal lymphoma in the modern radiation therapy era. Pract Radiat Oncol.

[63] Naimi Z, Moujahed R, Neji H (2021). Cardiac substructures exposure in left-sided breast cancer radiotherapy: is the mean heart dose a reliable predictor of cardiac toxicity?. Cancer Radiother.

[64] Eldesoky AR, Yates ES, Nyeng TB (2016). Internal and external validation of an ESTRO delineation guideline - dependent automated segmentation tool for loco-regional radiation therapy of early breast cancer. Radiother Oncol.

[65] Duane F, Aznar MC, Bartlett F (2017). A cardiac contouring atlas for radiotherapy. Radiother Oncol.

[66] Kaderka R, Gillespie EF, Mundt RC (2019). Geometric and dosimetric evaluation of atlas based auto-segmentation of cardiac structures in breast cancer patients. Radiother Oncol.

[67] Loap P, Tkatchenko N, Kirova Y (2020). Evaluation of a delineation software for cardiac atlas-based autosegmentation: An example of the use of artificial intelligence in modern radiotherapy. Cancer Radiother.

[68] Milo MLH, Offersen BV, Bechmann T (2020). Delineation of whole heart and substructures in thoracic radiation therapy: national guidelines and contouring atlas by the Danish Multidisciplinary Cancer Groups. Radiother Oncol.

[69] McWilliam A, Khalifa J, Vasquez Osorio E (2020). Novel methodology to investigate the effect of radiation dose to heart substructures on overall survival. Int J Radiat Oncol Biol Phys.

[70] Bruder H, Schaller S, Ohnesorge B, Mertelmeier T (1999) High-temporal-resolution volume heart imaging with multirow computed tomography. Proc. SPIE 3661, Medical Imaging: Image Processing. 10.1117/12.348597. Accessed 21 May 1999

[71] Kim S, Chang Y, Ra JB (2015). Cardiac motion correction based on partial angle reconstructed images in x-ray CT. Med Phys.

[72] Flohr TG, Bruder H, Stierstorfer K, Petersilka M, Schmidt B, McCollough CH (2008). Image reconstruction and image quality evaluation for a dual source CT scanner. Med Phys.

[73] Martin S, O’Brien R, Hofmann C, Keall P, Kipriditis J (2018). An in silico performance characterization of respiratory motion guided 4DCT for high-quality low-dose lung cancer imaging. Phys Med Biol.

[74] Morton N, Sykes J, Barber J, Hofmann C, Keall P, O'Brien R (2020). Reducing 4D CT imaging artifacts at the source: first experimental results from the respiratory adaptive computed tomography (REACT) system. Phys Med Biol.

